# Knowledge, attitude, and practice towards post-stroke osteoporosis among healthcare workers in Zhangjiakou, Hebei, China: Running title: KAP towards post-stroke osteoporosis

**DOI:** 10.1016/j.pmedr.2025.103300

**Published:** 2025-11-02

**Authors:** Fei Xue, Shuangshuang Xu, Jing Wang, Nan Xu, Yi Peng, Hong Shi, Xiaojuan Li

**Affiliations:** a Department of Health Management, The First Hospital of Zhangjiakou, Zhangjiakou, China; b Department of Gastroenterology, The First Hospital of Zhangjiakou, Zhangjiakou, China; c Department of Rehabilitation, The First Hospital of Zhangjiakou, Zhangjiakou, China; d Department of Endocrinology, Zhangjiakou First Hospital, Zhangjiakou, China; e Department of Equipment, Zhangjiakou Second Hospital, Zhangjiakou, China; f Department of Sports Medicine, Zhangjiakou Second Hospital, Zhangjiakou, China

**Keywords:** Stroke, Osteoporosis, Healthcare workers, Knowledge, attitude, and practice, Cross-sectional study

## Abstract

**Objective:**

This study aimed to investigate the knowledge, attitude, and practice (KAP) towards post-stroke osteoporosis among healthcare workers in Zhangjiakou City, Hebei, China.

**Methods:**

This cross-sectional study was conducted between January and April 2023 among healthcare workers in Zhangjiakou. Their demographic characteristics and KAP towards post-stroke osteoporosis were collected using a web-based questionnaire.

**Results:**

A total of 548 valid questionnaires were collected, their knowledge, attitude, and practice scores were 18 [14.0, 20.0] (possible range: 0–23), 30 [27.0, 34.0] (possible range: 7–35), and 37 [30.0, 43.0] (possible range: 10–50), respectively. Correlation analysis revealed positive relationships between knowledge and attitude (*r* = 0.49, *P* < 0.01), between attitude and practice (*r* = 0.56, P < 0.01), as well as between knowledge and practice (*r* = 0.45, P < 0.01).

**Conclusion:**

Healthcare workers in Zhangjiakou had a moderate knowledge, positive attitude, and moderate practice towards post-stroke osteoporosis. The study highlights the imperative to improve knowledge and promote positive attitude to enhance practice and improve patient outcomes in post-stroke osteoporosis management among healthcare workers.

## Highlights

• Investigated knowledge, attitude, and practice on post-stroke osteoporosis.
• 548 valid questionnaires showed moderate knowledge (18/23).
• Positive correlations between knowledge, attitude, and practice scores.
• Emphasizes need for enhanced education on post-stroke osteoporosis.

## 1 Introduction

Stroke is a major cerebrovascular disease (Herpich and Rincon, 2020). It stands as one of the leading causes of disability and mortality worldwide (Žilovič et al., 2021). Following stroke, individuals often encounter challenges in their recovery, including the development of secondary complications (Joundi and Menon, 2021). One such complication is post-stroke osteoporosis, a condition marked by decreased bone mineral density and an increased risk of fractures after a stroke (Wang et al., 2021). Physical inactivity and restricted mobility following a stroke contribute to muscle atrophy and bone loss, while factors like hormonal fluctuations, malnutrition, vitamin D deficiency, and medication use further exacerbate the risk.

The prevention and treatment of osteoporosis play a crucial role in the overall prognosis and rehabilitation of stroke patients. Osteoporosis-related fractures impede recovery, prolong hospitalization, and reduce quality of life (Wang et al., 2021). Therefore, assessing the knowledge, attitude, practice (KAP) of healthcare workers towards post-stroke osteoporosis is essential to identify areas for improvement. A KAP survey provides valuable insights into healthcare workers' understanding of the condition and their approach to preventive measures and treatment strategies (Alzghoul and Abdullah, 2015; Tahani and Manesh, 2021). Understanding healthcare workers' KAP is vital to guide interventions and improve care.

Despite existing studies evaluating healthcare workers' KAP levels towards osteoporosis (Mahdaviazad et al., 2018; Nohra et al., 2022), limited research focuses explicitly on post-stroke osteoporosis. Most existing work addresses osteoporosis in general rather than the specific context of post-stroke patients, leaving gaps in understanding healthcare workers' knowledge and practice in this area. Therefore, this study aimed to investigate the KAP towards post-stroke osteoporosis among healthcare workers. Understanding the current level of knowledge, attitude, and practice among healthcare workers is critical for identifying educational gaps and clinical priorities. The results of this study can help inform the development of targeted training programs, improve guideline adherence, and promote evidence-based interventions. Ultimately, enhancing knowledge and attitude in this area may contribute to reducing post-stroke fracture risk, optimizing rehabilitation outcomes, and shaping healthcare policy for more effective secondary prevention of osteoporosis.

## 2 Methods

### 2.1 Study design and participants

This web-based cross-sectional study was conducted among healthcare workers in Zhangjiakou between January and April 2023. Participants were recruited from 15 health and community institutions in Zhangjiakou through the Health Management Department, using convenience sampling. The inclusion criteria were: First, active healthcare workers currently employed in a healthcare setting; Second, participants self-reported their potential involvement in the management of post-stroke osteoporosis. The exclusion criteria were: First, healthcare workers in administrative or managerial positions outside of clinical practice; Second, questionnaires with incomplete or conflicting responses; Third, failure to correctly answer a predefined attention-check item designed for quality control. Direct experience was not required, only potential involvement. The study obtained ethical approval from the Ethics Committee of The Zhangjiakou First Hospital, and informed consent was obtained from all study participants.

### 2.2 Questionnaire and data collection

The questionnaire used in this study was developed based on relevant literature (Hsu et al., 2022; Kapoor et al., 2019). It was then refined with the input of two senior specialists, one with over 10 years of experience in orthopedics and another with over 10 years of experience in endocrinology. A pilot study was conducted among 25 participants, resulting in a Cronbach's α coefficient of 0.92, indicating good internal consistency.

The final questionnaire includes four dimensions: demographic characteristics, knowledge dimension, attitude dimension, and practice dimension. Additionally, a “trap question” which instructed participants to select a particular answer (e.g., “Please select option B") was set to confirm that they were reading attentively. This approach is commonly used in online surveys to identify careless or automated responses. The knowledge section assessed understanding of post-stroke osteoporosis including risk factors (e.g., immobility, hormonal use), clinical consequences (e.g., fracture risk), and management strategies (e.g., medications, screening tools). The attitude section included statements regarding the importance of prevention and monitoring in stroke patients, use of bone density tests, and treatment preferences. The practice section evaluated self-reported behaviors such as recommending diagnostic tests, prescribing supplements or medications, and educating patients about osteoporosis. The demographic characteristics dimension included gender, age, education, occupation, working experience, professional title, and department. The knowledge dimension consisted of a total of 13 questions with 24 items. The first 12 questions were scored on a binary scale, where a correct answer received one point, and an incorrect or unclear answer received zero points. The possible range for knowledge score was 0–23. Question 13 was designed for validity checking and was excluded from the total score. The attitude dimension included seven questions, assessed using a five-point Likert scale ranging from strongly agree (five points) to strongly disagree (one point), and the possible range for attitude score was 7–35. The practice dimension consisted of 10 questions, evaluated on a five-point Likert scale ranging from always (five points) to never (one point), and the possible range for practice score was 10–50. Participants' knowledge, attitude, and practice levels were categorized as inadequate, moderate, or adequate based on the percentage of the total score following Bloom's cut-off point (Kaliyaperumal, 2004). Specifically, a score of less than 60 % of the total was considered inadequate, 60 %–80 % as moderate, and above 80 % as adequate. Accordingly, for the knowledge section (total score: 23), scores <13.80 were categorized as inadequate, 13.80–18.40 as moderate, and > 18.40 as adequate. For the attitude section (total score: 35), scores <21 were considered negative, 21–28 as moderate, and > 28.0 as positive. For the practice section (total score: 50), scores <30 were regarded as insufficient, 30–40 as moderate, and > 40 as sufficient.

The electronic questionnaire *“Sojump (**www.wjx.cn**)”* was distributed via the Health Management Department, accessed through link or quick response code.

### 2.3 Statistical analysis

Before conducting the formal analysis, the minimum required sample size was estimated using Cochran's formula for proportions:

$$n=Z^{2}\times P\times \left(1-P\right)/d^{2}$$

where Z is the standard normal variate (1.96 for a 95 % confidence level), P is the expected proportion of adequate knowledge (assumed to be 50 % due to a lack of prior data), and d is the margin of error (set at 5 %). The calculated minimum sample size was 384. The normality of continuous variables was tested using the Shapiro–Wilk test, and all variables were found to be non-normally distributed (*P* < 0.01). Therefore, continuous data are presented as median with interquartile range [Q1, Q3], and group comparisons were conducted using non-parametric tests (Mann–Whitney *U* test or Kruskal–Wallis H test as appropriate). Categorical data were presented as n (%). Pearson analysis was applied to analyze the correlation between knowledge, attitude, and practice scores. A two-sided *P* < 0.05 was considered statistically significant. Statistical Product and Service Solutions version 26.0 (IBM, Armonk, New York, USA) was used for statistical analysis.

## 3 Results

A total of 1614 questionnaires were received, and after screening to eliminate invalid questionnaires based on the attention-check question, missing data, and self-reported potential involvement in post-stroke osteoporosis management, 548 valid questionnaires were included for analysis. The final 548 participants exceeded the required sample size, ensuring statistical power. Among the 548 participants, 182 (33.21 %) were male, and 366 (66.79 %) were female. The age distribution was approximately evenly split, with 52.74 % falling between 20 and 40 years old and 47.26 % being 40 years and above. In terms of education, 50.73 % had a junior college education or below, while 49.27 % held a bachelor's degree or above. Most of the participants 410 (74.82 %) came from departments that were not directly involved in management of patients with stroke (Table 1). Among the 548 participants, based on the Bloom's cut-off criteria, 242 (44.16 %) were classified as having high knowledge, 210 (38.32 %) as moderate, and 96 (17.52 %) as low. For attitude, 326 (59.49 %) demonstrated a positive level, 211 (38.50 %) a moderate level, and 11 (2.01 %) a negative level. Regarding practice, 183 (33.39 %) exhibited high-level practice, 224 (40.88 %) moderate, and 141 (25.73 %) low.

**Table 1:** Demographic characteristics and knowledge, attitude, and practice scores of healthcare workers in Zhangjiakou, China, January–April 2023.

Variables	N (%)	Knowledge (possible range: 0–23)	P	Attitude (possible range: 7–35)	P	Practice (possible range: 10–50)	P
Total							
Gender			0.04		0.03		0.04
Male	182(33.21)	18(15.0,21.0)		31(28.0,35.0)		38(32.0,45.0)	
Female	366(66.79)	18(14.0,20.0)		30(27.0,34.0)		36(30.0,42.0)	
Age, years			0.18		0.45		0.69
20–40	289(52.74)	18(15.0,21.0)		30(27.0,35.0)		37(30.0,43.0)	
≥ 40	259(47.26)	18(14.0,22.0)		30(27.0,34.0)		37(31.0,44.0)	
Education			<0.01		<0.01		0.65
Junior college and below	278(50.73)	18(14.0,20.0)		29(27.0,34.0)		37(31.0,43.0)	
Bachelor's degree and above	270(49.27)	18(16.0,21.0)		31(28.0,35.0)		37(30.0,44.0)	
Occupation			<0.01		0.01		0.24
Doctor	257(46.90)	19(16.0,21.0)		31(28.0,35.0)		37(31.0,44.5)	
Nurse	15(29.01)	17(14.0,20.0)		29(27.0,34.0)		36(30.0,41.0)	
Medical technician	77(14.05)	17(13.0,20.0)		30(27.0,34.0)		38(32.0,42.5)	
Others	55(10.04)	17(13.0,19.0)		28(26.0,33.0)		36(30.0,48.0)	
Working experience, years			0.14		0.77		0.35
0–5	142(25.91)	18(15.8,21.0)		30(27.0,35.0)		38(31.0,44.3)	
6–10	89(16.24)	18(14.5,20.5)		30(27.0,35.0)		37(30.0,44.5)	
11–15	89(16.24)	19(15.0,20.5)		29(27.0,34.5)		36(27.0,41.0)	
≥16	228(41.61)	17(14.0,20.0)		30(28.0,34.0)		37(31.0,43.0)	
Professional title			<0.01		0.02		0.12
Junior	217(39.60)	18(15.0,21.0)		30(28.0,35.0)		38(31.0,42.5)	
Intermediate	142(25.91)	17(14.0,20.0)		30(27.0,34.0)		35(29.0,41.3)	
Senior	68(12.41)	19(17.0,21.0)		31(28.0,35.0)		39(33.0,47.8)	
None	121(22.08)	17(14.0,20.0)		29(27.0,34.0)		37(30.0,45.0)	
Department			0.10		0.03		0.19
Neurology and Neurosurgery	36(6.57)	19(18.0,21.0)		33(29.0,35.0)		41(35.0.25,5)	
Geriatrics	11(2.01)	17(14.0,21.0)		31(27.0,35.0)		34(26.0,40.0)	
Rehabilitation	31(5.66)	20(15.0,20.0)		31(27.0,35.0)		37(31.0,41.0)	
Orthopedics	25(4.56)	17(15.0,20.5)		29(26.5,34.0)		35(30.0,41.0)	
Endocrinology	9(1.64)	19(10.5,21.0)		32(26.0,35.0)		40(24.5,45.0)	
Medical Imaging	26(4.74)	18(14.8,21.0)		33(28.0,35.0)		40(31.0,44.0)	
Others	410(74.82)	18(14.0,20.0)		29(27.0,34.0)		37(30.0,43.0)	

Footnote:

Values: Values are presented as median [Q1, Q3] (first quartile, third quartile).

Statistical Tests: *P*-values were generated using the Mann-Whitney U test and Kruskal-Wallis H test as appropriate.

Professional title: Junior – holders of junior professional titles (e.g., resident physician, junior nurse/technologist); Intermediate – holders of intermediate professional titles (e.g., attending physician, intermediate nurse/technologist); Senior – holders of senior professional titles (e.g., associate chief/chief physician; associate senior/senior nurse/technologist); None – no formal professional title.

**Fig. 1:**
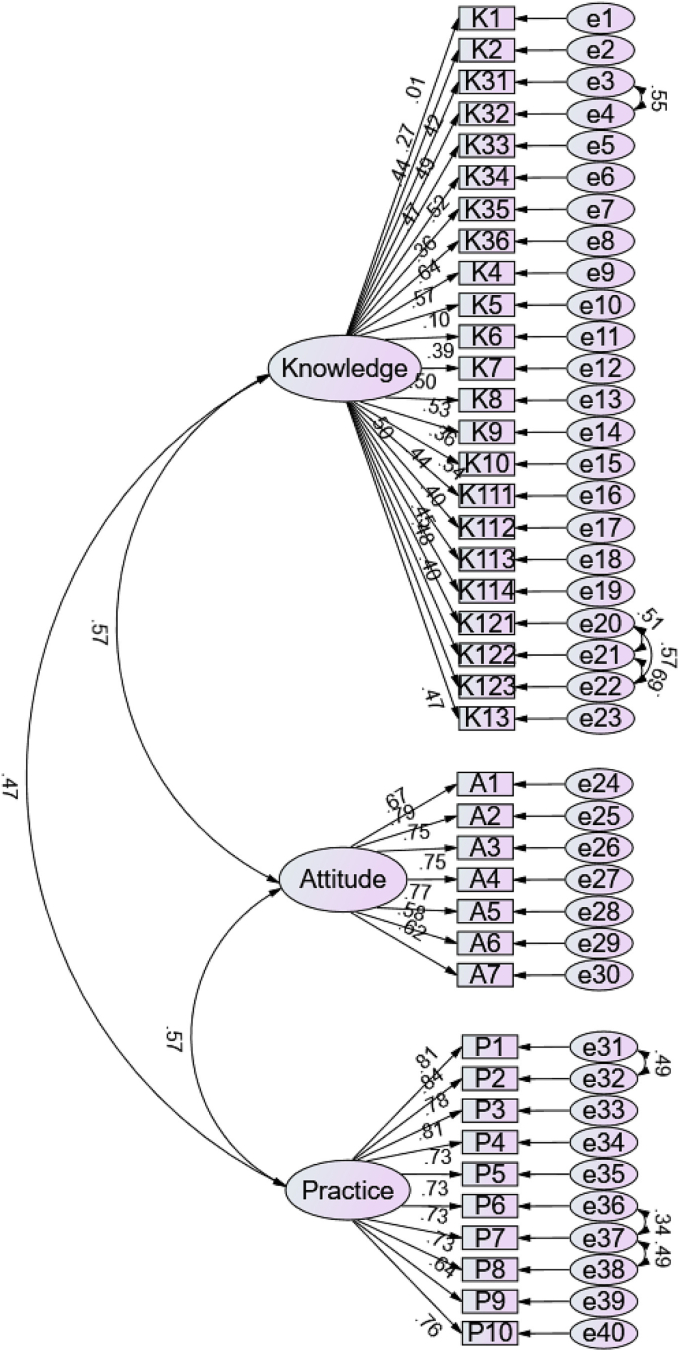
Confirmatory factor analysis model of knowledge, attitude, and practice among healthcare workers in Zhangjiakou, China, January–April 2023. Footnote: The rectangles represent observed variables (questionnaire items), and the ellipses represent latent constructs. The arrows indicate factor loadings, and e1–en represent measurement error terms associated with each observed variable. All factor loadings are significant (*P* < 0.01), and model fit indices indicate acceptable fit (Kaiser-Meyer-Olkin = 0.91, Chi-Square Minimum/Degrees of Freedom = 2.92, Root Mean Square Error of Approximation = 0.06, Incremental Fit Index = 0.86, Tucker-Lewis Index = 0.85, Comparative Fit Index = 0.86).

A post hoc confirmatory factor analysis (CFA) was performed using the data collected in the formal survey to assess construct validity. The Kaiser-Meyer-Olkin value was 0.91 (*P* < 0.01), indicating sampling adequacy. Model fit indices suggested acceptable fit (Chi-Square Minimum/Degrees of Freedom = 2.92, Root Mean Square Error of Approximation = 0.06, Incremental Fit Index = 0.86, Tucker-Lewis Index = 0.85, Comparative Fit Index = 0.86), supporting a three-factor structure consistent with the knowledge, attitude, and practice dimensions. The CFA results are shown in Fig. 1.

In the knowledge dimension, the participants exhibited a moderate knowledge with a score of 18 [14.0, 20.0] (possible range: 0.0–23.0). Participants who were male, held a bachelor’ s degree or above, were physicians, or held higher professional titles tended to have higher knowledge scores (Table 1). The question *“All elderly people suffer from osteoporosis”* had the lowest correct rate (38.87 %). Conversely, the question *“Lack of sun exposure or dietary deficiencies in calcium/vitamin D might increase the risk of disease”* had the highest correct rate (97.26 %) (Supplementary table 1).

In the attitude dimension, participants demonstrated an overall positive attitude, with a score of 30[27.0, 34.0] (possible range: 7.0–35.0). The attitude scores showed significant difference among participants with different gender, education, occupation, professional title, and department (Table 1). The question *“In patients with post-stroke dysphagia, the drug can be administered intravenously”* received the lowest score, indicating an area where participants showed less favorable attitude. Conversely, the question *“Stroke patients need to be prevented from developing osteoporosis better than healthy people”* received the highest score, indicating a strong positive attitude among the participants (Supplementary table 2).

In the practice dimension, participants demonstrated a moderate practice, with a score of 37 [30.0, 43.0] (possible range: 10.0–50.0), and male participants showed higher score (Table 1). The question about *“Treated with Denosumab"* had the lowest score, indicating an area where participants exhibited less optimal practice. On the other hand, *“educating stroke patients about osteoporosis prevention”* received the highest score, indicating a well-implemented practice among the participants (Supplementary table 3).

The Pearson correlation analysis revealed significant positive relationships between knowledge and attitude (*r* = 0.49, *P* < 0.01), attitude and practice (*r* = 0.56, P < 0.01), as well as knowledge and practice (*r* = 0.45, P < 0.01) (Table 2).

**Table 2:** Correlations between knowledge, attitude, and practice among healthcare workers in Zhangjiakou, China, January–April 2023.

	Knowledge	Attitude	Practice
Knowledge	1		
Attitude	0.49 (P < 0.01)	1	
Practice	0.45 (P < 0.01)	0.56 (P < 0.01)	1

## 4 Discussion

This cross-sectional study revealed that healthcare workers demonstrated a moderate level of knowledge, positive attitude, and moderate practice towards post-stroke osteoporosis. This underscores the need to enhance knowledge and attitude to improve practice. Implementing targeted interventions that promote knowledge and foster positive attitude can empower healthcare workers to enhance their practice in post-stroke osteoporosis management, leading to improved patient outcomes.

Our findings are consistent with prior research reporting moderate levels of knowledge and practice among healthcare workers regarding osteoporosis management (Mahdaviazad et al., 2018)(Chan et al., 2019). This suggests that KAP levels may be similar across healthcare settings and regions.

Some studies have reported different KAP levels, with higher scores in rehabilitation settings and lower scores in geriatric populations (Tay et al., 2022)(Day et al., 2018)(Hallit et al., 2019). Such discrepancies may reflect variations in study populations, cultural factors, healthcare systems, and access to educational resources (Chelf et al., 2022)(Chan et al., 2018).

In the present study, participants exhibited moderate levels of knowledge, with male participants and those with higher education levels obtaining higher scores in the knowledge and attitude dimensions. These findings are consistent with previous studies that have reported similar gender and educational discrepancies in knowledge levels. For instance, Chan et al. found that male healthcare workers scored higher on knowledge assessments related to osteoporosis compared to their female counterparts (CY, Subramaniam, Ravintharan, Ima-Nirwana, & Chin, 2021), whereas other researchers have reported the opposite outcome (Ishtaya, Anabtawi, Zyoud, & Sweileh, 2018; Wong et al., 2013). Likewise, Carolina et al. conducted a study revealing that healthcare workers with higher education levels demonstrated better knowledge about osteoporosis management (Day et al., 2018). These results underscore the necessity for targeted educational interventions to address these knowledge disparities. Additionally, physicians and those with higher professional titles also demonstrated significantly higher knowledge scores. This may be due to greater clinical exposure, more decision-making responsibilities, and increased access to training or academic resources often associated with these roles. These findings align with prior studies showing that male healthcare professionals often demonstrate higher KAP scores, especially in knowledge and clinical decision-making domains, potentially due to greater self-efficacy and more extensive experience(Romem and Rozani, 2024). Moreover, higher educational attainment is frequently associated with improved access to structured continuing medical education and professional development, which has been shown to significantly enhance both knowledge and practical competence in clinical settings(Samuel et al., 2021). However, in our study, while educational level was significantly associated with higher knowledge and attitude scores, it was not associated with differences in practice scores. This suggests that improvements in knowledge and attitude may not automatically lead to better clinical practice. Additional structural or behavioral interventions may be required to translate awareness into consistent action. Implementing educational programs or workshops specifically tailored to enhance knowledge among female healthcare workers or those with lower education levels could help bridge these gaps and promote improved patient care.

The participants' positive attitude towards post-stroke osteoporosis management, as seen in previous research demonstrating positive attitude among healthcare professionals in various settings (Khan et al., 2019; Mahdaviazad et al., 2018; Peng et al., 2020), have implications for the quality of care provided to stroke survivors. These positive attitude may stem from sufficient awareness and understanding of the importance of managing post-stroke osteoporosis and its impact on the overall health and well-being of stroke survivors. Educational interventions could further improve attitude. Additionally, contextual factors such as heavy workloads, time constraints, and competing priorities may contribute to diminished motivation or the ability to prioritize this aspect of stroke care (Mahdaviazad et al., 2018). Addressing these systemic issues, providing support, and allocating adequate resources to healthcare workers are essential in fostering a more positive attitude towards post-stroke osteoporosis management.

The findings from the practice section of the study reveal an overall moderate level of post-stroke osteoporosis management practice among the participants. This highlights the need to improve care quality. Interestingly, no significant differences in scores were observed based on demographic factors, suggesting a consistent lack of practice across different groups. Addressing specific weaknesses is crucial to enhance practice. This study emphasizes the significance of targeted interventions, interdisciplinary collaborations, and policy changes to bridge the gap between knowledge and practice, which is in line with previous research highlighting the challenges in implementing evidence-based practices in healthcare (Evans-Lacko et al., 2010).

The clinical significance of this study lies in its implications for post-stroke osteoporosis management. By recognizing the moderate levels of knowledge and practice among healthcare workers, along with the presence of negative attitude, targeted interventions can be developed to address the identified gaps. Educational programs should concentrate on enhancing knowledge and promoting positive attitude towards post-stroke osteoporosis management (Carda et al., 2009). These efforts may improve practice and outcomes. Training curricula could cover Denosumab, early screening tools (Fracture Risk Assessment Tool, Osteoporosis Self-Assessment Tool for Asians), and exercise-based prevention. To further validate the effectiveness of such educational strategies, future longitudinal or interventional studies are recommended to assess whether improvements in healthcare workers' KAP actually translate into reduced fracture incidence among post-stroke patients.

This study has several limitations. It was conducted at a single center in Zhangjiakou City with a relatively small sample, which may limit generalizability. The valid response rate was reduced by strict quality control, potentially introducing selection bias. This high exclusion rate may also reduce external validity by limiting the representativeness of the final sample. The cross-sectional design also precludes causal inference. Data were collected between January and April 2023, and more recent guidelines such as the 2023 Osteoporosis Canada recommendations may influence applicability in some settings. Self-report measures and online survey methods may have introduced recall or selection bias. In particular, self-selected participants may have higher baseline knowledge and attitudes than the general healthcare worker population, and certain specialties were underrepresented. Future research should include larger, more diverse samples and longitudinal or interventional designs to validate and extend these findings.

In conclusion, this study reveals that healthcare workers in Zhangjiakou City exhibit moderate knowledge, positive attitude, and moderate practice towards post-stroke osteoporosis. Educational intervention is essential to improve practical application and ultimately enhance patient outcomes. Further research is necessary to address disparities and assess the impact of enhancing KAP on clinical outcomes.

## CRediT authorship contribution statement

**Fei Xue:** Writing – original draft, Conceptualization. **Shuangshuang Xu:** Data curation, Conceptualization. **Jing Wang:** Formal analysis. **Nan Xu:** Project administration, Methodology. **Yi Peng:** Writing – review & editing. **Hong Shi:** Visualization, Validation. **Xiaojuan Li:** Visualization.

## Consent for publication

Not applicable.

## Ethics approval and consent to participate

The study obtained ethical approval from the Ethics Committee of The Zhangjiakou First Hospital, and informed consent was obtained from all study participants. I confirm that all methods were performed in accordance with the relevant guidelines. All procedures were performed in accordance with the ethical standards laid down in the 1964 Declaration of Helsinki and its later amendments.

## Funding

This research did not receive any specific grant from funding agencies in the public, commercial, or not-for-profit sectors.

## Declaration of competing interest

The authors declare that they have no known competing financial interests or personal relationships that could have appeared to influence the work reported in this paper.

## Data Availability

No data was used for the research described in the article.

## Appendix A Supplementary data

Supplementary material Image 1
