# Supplementary material for: Knowledge, attitude, and practice towards post-stroke osteoporosis among healthcare workers in Zhangjiakou, Hebei, China: Running title: KAP towards post-stroke osteoporosis
Source: Prev Med Rep. 2025 Nov 2;60:103300. doi: 10.1016/j.pmedr.2025.103300 (PMC12664384; doi:10.1016/j.pmedr.2025.103300)
Supplement: Supplementary file 1 — Supplementary material [file mmc1.docx]

**Supplementary Table 1.** Knowledge items and response distributions among healthcare workers in Zhangjiakou, China, January–April 2023

| Knowledge | N (%) | |
| --- | --- | --- |
|  | Wrong | Correct |
| 1. All elderly people suffer from osteoporosis | 335(61.13) | 213(38.87) |
| 2. Osteoporosis is usually asymptomatic in its early stages | 124(22.63) | 424(77.37) |
| 3. Which of the following are risk factors for osteoporosis |  |  |
| Smoking | 172(31.39) | 376(68.61) |
| Alcohol consumption | 119(21.72) | 429(78.28) |
| Lack of sun exposure or dietary deficiencies in calcium/vitamin D | 15(2.74) | 533(97.26) |
| Lack of exercise, e.g. prolonged bed rest | 19(3.47) | 529(96.53) |
| Long-term hormone use (e.g. glucocorticoids) | 43(7.85) | 505(92.15) |
| Low body weight | 310(56.57) | 238(43.43) |
| 3. Patients with stroke are more likely to develop osteoporosis because of the paralysis, reduced exercise and reduced bone load that result from stroke | 68(12.41) | 480(87.59) |
| 4. The use of medication during stroke treatment (e.g. warfarin, anti-epileptic drugs) may lead to a reduction in bone density | 116(21.17) | 432(78.83) |
| 5. Osteoporosis is not preventable in stroke patients | 223(40.69) | 325(59.31) |
| 6. Age and gender are risk factors for the development of osteoporosis after stroke | 211(38.5) | 337(61.5) |
| 7. Osteoporosis causes a decrease in bone mass and an increase in bone fragility, which predisposes to fracture | 17(3.1) | 531(96.9) |
| 8. Falls and fractures are one of the main factors affecting the recovery and quality of life of stroke patients | 48(8.76) | 500(91.24) |
| 9. Rehabilitation can be effective in preventing post-stroke osteoporosis | 74(13.5) | 474(86.5) |
| 10. Do you know whether the following types of drugs are available for the treatment of osteoporosis |  |  |
| Drugs that inhibit osteoclasts (e.g. dihydrophosphonates) | 201(36.68) | 347(63.32) |
| Drugs that inhibit bone resorption (e.g. calcitonin) | 166(30.29) | 382(69.71) |
| Hormonal drugs (e.g. oestrogen/progestin, parathyroid hormone) | 206(37.59) | 342(62.41) |
| Antibody drugs (e.g. Denosumab) | 252(45.99) | 296(54.01) |
| 11. Do you know the following easy screening tools for osteoporosis |  |  |
| Fracture Risk Assessment Tool (FRAX) | 169(30.84) | 379(69.16) |
| one-minute quiz for osteoporosis of International Osteoporosis Foundation (IOF) | 169(30.84) | 379(69.16) |
| Osteoporosis Self-Assessment Tool for Asians (OSTA) | 159(29.01) | 389(70.99) |
| 12. Osteoporosis can be prevented or treated by improving light and restructuring the diet | 23(4.2) | 525(95.8) |

**Supplementary Table 2.** Attitude items and response distributions among healthcare workers in Zhangjiakou, China, January–April 2023

| Attitude | Strongly agree | Agree | Neutral | Disagree | Strongly disagree |
| --- | --- | --- | --- | --- | --- |
| 1. Stroke patients need to be prevented from developing osteoporosis better than healthy people | 335(61.13) | 181(33.03) | 26(4.74) | 5(0.91) | 1(0.18) |
| 1. Stroke patients should have regular bone density testing | 298(54.38) | 215(39.23) | 29(5.29) | 3(0.55) | 3(0.55) |
| 1. The risk-benefit ratio should be taken into account in the selection of medication for the treatment of the elderly patients with post-stroke osteoporosis | 293(53.47) | 212(38.69) | 34(6.2) | 9(1.64) | - |
| 1. Regular bone density testing is also recommended for young stroke patients | 239(43.61) | 237(43.25) | 60(10.95) | 10(1.82) | 2(0.36) |
| 1. The supplementation of Vitamin D and calcium in stroke patients should be considered in combination with the test results of bone metabolism marker | 256(46.72) | 251(45.8) | 38(6.93) | 3(0.55) | - |
| 1. In patients with post-stroke dysphagia, the drug can be administered intravenously | 189(34.49) | 233(42.52) | 91(16.61) | 29(5.29) | 6(1.09) |
| 1. Combination of multiple drugs may improve the prevention/treatment of osteoporosis | 208(37.96) | 203(37.04) | 94(17.15) | 38(6.93) | 5(0.91) |

**Supplementary Table 3.** Practice items and response distributions among healthcare workers in Zhangjiakou, China, January–April 2023

| Practice | Always | Often | Usually | Occasionally | Never |
| --- | --- | --- | --- | --- | --- |
| 1. I would recommend regular bone density testing for stroke patients | 158(28.83) | 227(41.42) | 90(16.42) | 56(10.22) | 17(3.1) |
| 2. I would recommend regular bone metabolism markers testing for stroke patients | 157(28.65) | 194(35.4) | 109(19.89) | 58(10.58) | 30(5.47) |
| 3. I would recommend vitamin D or calcium supplementation based on the results of bone metabolism markers testing | 206(37.59) | 225(41.06) | 70(12.77) | 33(6.02) | 14(2.55) |
| 4. I would educate stroke patients about osteoporosis prevention | 232(42.34) | 199(36.31) | 57(10.4) | 47(8.58) | 13(2.37) |
| 5. I would recommend additional medication to prevent osteoporosis in stroke patients who are already on multiple medications | 142(25.91) | 146(26.64) | 129(23.54) | 78(14.23) | 53(9.67) |
| 6. I would recommend intravenous bisphosphonates for the prevention of osteoporosis in stroke patients who are already on multiple oral medications | 113(20.62) | 114(20.8) | 136(24.82) | 101(18.43) | 84(15.33) |
| 7. I would recommend that patients with stroke are treated with Denosumab | 108(19.71) | 112(20.44) | 128(23.36) | 95(17.34) | 105(19.16) |
| 8. I would advise patients who are being treated with bisphosphonates to discontinue it while treated with Denosumab | 121(22.08) | 118(21.53) | 139(25.36) | 86(15.69) | 84(15.33) |
| 9. I would recommend that patients with stroke have regular rehabilitative treatment | 240(43.8) | 208(37.96) | 58(10.58) | 27(4.93) | 15(2.74) |
| 10. I would recommend that stroke patients have at least one self-test for osteoporosis (e.g. IOF, OSTA) | 203(37.04) | 195(35.58) | 77(14.05) | 41(7.48) | 32(5.84) |
